# Use of Clodronate Liposomes to Deplete Phagocytic Immune Cells in *Drosophila melanogaster* and *Aedes aegypti*

**DOI:** 10.3389/fcell.2021.627976

**Published:** 2021-02-02

**Authors:** Jyothsna Ramesh Kumar, Jessica P. Smith, Hyeogsun Kwon, Ryan C. Smith

**Affiliations:** ^1^Interdepartmental Graduate Program in Immunobiology, Iowa State University, Ames, IA, United States; ^2^Department of Entomology, Iowa State University, Ames, IA, United States

**Keywords:** phagocytosis, hemocytes, immune cells, phagocyte depletion, clodronate liposomes, *Aedes (Ae.) aegypti*, *Drosophila melanogaster*

## Abstract

The innate immune system is the primary defense response to limit invading pathogens for all invertebrate species. In insects, immune cells are central to both cellular and humoral immune responses, however few genetic resources exist beyond *Drosophila* to study immune cell function. Therefore, the development of innovative tools that can be widely applied to a variety of insect systems is of importance to advance the study of insect immunity. Here, we have adapted the use of clodronate liposomes (CLD) to deplete phagocytic immune cells in the vinegar fly, *Drosophila melanogaster*, and the yellow fever mosquito, *Aedes aegypti*. Through microscopy and molecular techniques, we validate the depletion of phagocytic cell populations in both insect species and demonstrate the integral role of phagocytes in combating bacterial pathogens. Together, these data demonstrate the wide utility of CLD in insect systems to advance the study of phagocyte function in insect innate immunity.

## Graphical Abstract

Overview of phagocyte depletion experiments using clodronate liposomes to in *Drosophila melanogaster* or *Aedes aegypti*. Created with BioRender.com. 
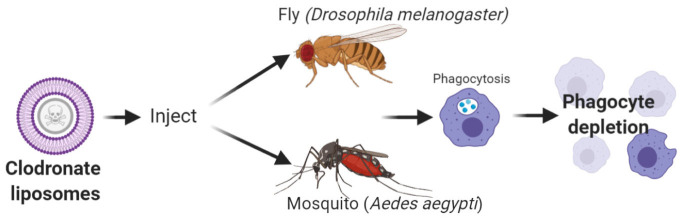

## Introduction

Insects rely on conserved cellular and humoral responses as the primary defense to invading pathogens. Immune cells, known as hemocytes, can directly participate in cellular responses such as phagocytosis and encapsulation (Lemaitre and Hoffmann, 2007; Hillyer and Strand, 2014), as well as mediate humoral signaling responses (Foley and O’Farrell, 2003; Wu et al., 2012) that limit pathogen survival. Studies in *Drosophila* have been aided by a wealth of genetic tools that include mutant and transgenic lines (Braun et al., 1997, 1998; Kurucz et al., 2003; Zettervall et al., 2004), as well as genetic techniques to ablate populations of plasmatocytes (Charroux and Royet, 2009; Defaye et al., 2009) that have significantly advanced our understanding of insect immune cells. However, the lack of genetic resources in non-model insect systems has severely limited studies of immune cell function. In mosquitoes, there has been a dependence on RNAi for reverse-genetic studies of hemocytes (Pinto et al., 2009; Ramirez et al., 2014; Smith et al., 2015, 2016), yet due to the absence of hemocyte markers and the systemic nature of gene-silencing, there have been significant limitations to address gene function in specific tissues or immune cell-types.

Evidence from vertebrate systems has demonstrated that chemical approaches can be utilized to target immune cells (Shek and Lukovich, 1982; Kagan and Hartmann, 1984; van Rooijen and Sanders, 1994), overcoming specific requirements for genetic tools to study immune cell function. Among these chemical approaches, clodronate liposomes (CLD) have shown the most promise and have been widely used in vertebrate systems to examine macrophage function (van Rooijen and Sanders, 1994; Lehenkari et al., 2002; van Rooijen and Hendrikx, 2010). Relying on the phagocytic properties of a subset of immune cells, CLD can be specifically delivered to macrophage populations, where after being phagocytosed they are degraded by the lysosome to promote apoptosis (van Rooijen and Sanders, 1994; van Rooijen and Hendrikx, 2010). Non-target cells lacking phagocytic abilities and lysosomal components are not affected by CLD treatment (van Rooijen and Sanders, 1994; van Rooijen and Hendrikx, 2010). This methodology has been widely applied in mammalian systems to understand autoimmune disease and macrophage contributions to infection biology (Jordan et al., 2003; Cockburn et al., 2010; Cha et al., 2015).

A recent study in mosquitoes described the use of CLD to deplete phagocytic immune cell populations in *Anopheles gambiae* (Kwon and Smith, 2019), demonstrating for the first time that CLD can be utilized in an invertebrate. Based on the highly conserved phagocytic properties of immune cells, the use of CLD has significant potential as a tool to study invertebrate immune function, overcoming many of the technical hurdles for non-model insect species. To further examine its applicability to insect species, in this study we examine the use of CLD to similarly investigate phagocytic immune cell function in *Drosophila melanogaster* and *Aedes aegypti*. Through these studies, we demonstrate that CLD can effectively deplete phagocytic cell populations of both species, illustrating the broad application of the use of CLD to study innate immune cell function across insects.

## Methods

### Fly Stocks

*Drosophila melanogaster* fly stocks were maintained at 25°C on standard molasses-based fly medium (Archon Scientific). Previously described SRP-mCherry (w[1118]; P{w[ + mC] = srpHemo-3XmCherry}; stock #78358) and HeGal4-UAS-GFP (w[^∗^]; P{w[ + mC] = He-GAL4.Z}85, P{w[ + mC] = UAS-GFP.nls}8; stock #8700) transgenic lines (Zettervall et al., 2004; Gyoergy et al., 2018) which express fluorescent proteins under universal larval hemocyte markers were obtained from the Bloomington Stock Center.

### Mosquito Rearing

*Aedes aegypti* (Liverpool strain) mosquitoes were reared at 27°C and 80% relative humidity with a 14:10 h light/dark period. Larvae were reared on a 50:50 diet of ground fish flakes (Tetramin, Tetra) and milk bone dog biscuits. Adults were maintained on a 10% sucrose solution. All experimental techniques were performed on cohorts of 4–6 days old adult female mosquitoes.

### Phagocytic Cell Depletion Using Clodronate Liposomes

Adult flies (2–3 days old) and mosquitoes (3–5 days old) were intra-thoracically injected with 69 nl of control liposomes (LP) or CLD (Standard macrophage depletion kit, Encapsula NanoSciences LLC) using a Nanoject III injector (Drummond Scientific) as previously described (Kwon and Smith, 2019). To determine the ideal concentrations for each species to maximize CLD efficacy on phagocyte depletion while minimizing effects on survival, dilutions of commercially available stock solutions of LP (24.3 mM L-alpha-phosphatidylcholine, 10.9 mM cholesterol) and CLD (24.3 mM L-alpha-phosphatidylcholine, 10.9 mM cholesterol, 18.4 mM Clodronate [(Dichloro-phosphono-methyl)phosphonate) were prepared in 1X PBS (1 (stock), 1:2, 1:3, 1:4 (only *Aedes*), 1:5] and compared to 1× PBS serving as an injection control. Based on the resulting experiments, a 1:5 dilution was chosen for all subsequent experiments in *Drosophila*, while a 1:4 dilution of LP and CLD was used for experiments with *Ae. aegypti*.

### Hemolymph Perfusion and Counting of Hemocytes

To evaluate the efficacy of phagocyte depletion experiments, hemolymph perfusions were performed as previously (Smith et al., 2015; Kwon et al., 2017; Kwon and Smith, 2019) using anticoagulant buffer (vol/vol 60% Schneider’s insect medium, 10% fetal bovine serum and 30% citrate buffer, 98 mM NaOH, 186 mM NaCl, 1.7 mM EDTA, 41 mM citric acid, pH 4.5). Perfused hemolymph was placed onto a hemocytometer (Neubauer, C-Chip DHC-N01, INCYTO) where approximately 50 cells were counted per individual fly or approximately 200 cells per individual mosquito for both LP and CLD treated sub-groups. Hemocyte sub-populations were differentiated by morphology (size and shape) or fluorescence (red or green) in the *Drosophila* transgenic lines.

*Drosophila* samples were examined 48h post-injection, while *Aedes* were evaluated at both 24 and 48h post-injection. Additionally, to examine the effects of blood feeding, blood-fed mosquitoes were examined 24 h post blood-meal (48 h post-injection) after challenge with defibrinated sheep blood (Hemostat Laboratories) using an artificial membrane feeding system.

### Bacterial Challenge Following Clodronate Treatment

Cultures of *Serratia marcesens* and *Staphylococcus aureus* were grown overnight in LB at 37°C. For *Drosophila* experiments, bacterial cultures were centrifuged at 8,000 rpm for 5 min, washed twice with 1× PBS, and resuspended in 1× PBS at a concentration of OD_600_ = 0.1. Approximately 24 h after pre-treatment with LP or CLD, adult SRP-mCherry *Drosophila* (*n* = 20 per replicate) were injected with 23 nl (∼1 × 10^8^ CFU/ml) of either bacterial suspensions (*S. marcescens* or *S. aureus*) using a Nanoject III injector as previously described (Troha and Buchon, 2019). Following challenge, flies were maintained at room temperature and survival was monitored every 24 h for 8 days.

For mosquito experiments, *S. marcescens* or *S. aureus* cultures were centrifuged at 8,000 rpm for 5 min, washed twice with 1× PBS and resuspended to a final concentration of OD_600_ = 0.4. OD. A 100× dilution of the bacterial cultures (∼4 × 10^6^ CFU/ml) were injected (69 nl) into naïve adult mosquitoes (*n* = 30 per replicate) 48 h post-treatment with LP or CLD as previously (Kwon and Smith, 2019). The injection of 1× PBS was included as an additional control. The survival of mosquitoes following bacterial challenge was monitored every 24 h for 8 days to determine the effects of phagocyte depletion on mosquito survival.

### Gene Expression Analysis Following Clodronate Treatment

Total RNA was isolated from pooled whole fly or mosquito samples using TRIzol (Thermo Fisher Scientific), of which 2 μg of total RNA was used a template for cDNA synthesis using the RevertAid First Strand cDNA Synthesis kit (Thermo Fisher Scientific). To examine gene expression following phagocyte depletion, qRT-PCR was performed using PowerUp SYBR Green Master Mix (Thermo Fischer Scientific) on control- or clodronate-treated treated fly and mosquito samples.

To validate phagocyte depletion in *Drosophila*, primers directed at either GFP or mCherry were examined in their respective transgenic lines using RpL32 as an internal control (Supplementary Table 1) using the following cycling conditions: 95°C for 10 min, 40 cycles with 95°C for 15 s and 65°C for 60 s. Similarly, phagocyte depletion was evaluated in *Aedes* using primers directed at the granulocyte-enriched genes, nimrod, and eater, with rpS17 as an internal control (Supplementary Table 1). qRT-PCR was performed for 40 cycles using the following cycling conditions: 98°C for 10s, 60°C for 10s and 72°C for 30 s. For both fly and mosquito samples, relative expression was evaluated using a comparative C_T_ (2^–ΔΔCt^) method (Livak and Schmittgen, 2001).

## Results

**FIGURE 1:**
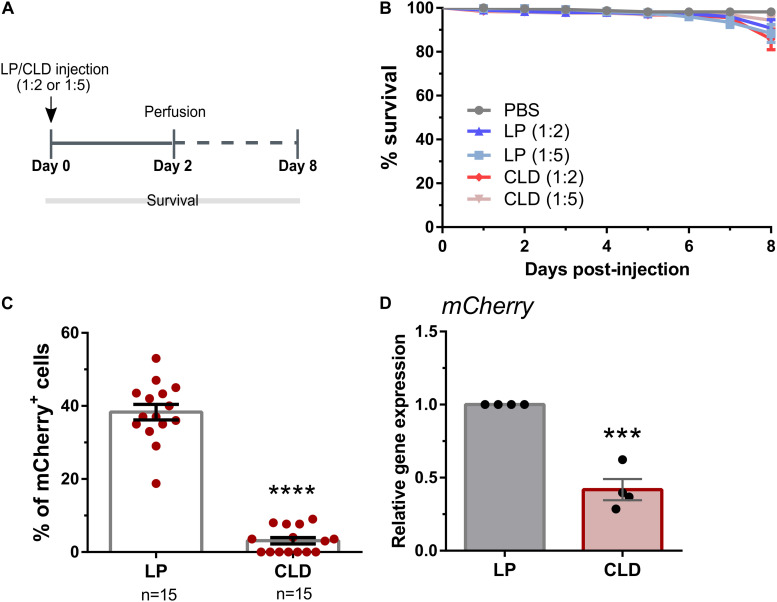
Use of clodronate liposomes to deplete *Drosophila* plasmatocytes. **(A)** Overview of clodronate liposome experiments in *Drosophila* (SRP-mCherry). Control (LP)- or clodronate liposomes (CLD) were diluted at 1:2 or 1:5 in 1X PBS and intrathoracically injected into adult female flies. Survival was then monitored over an eight-day period **(B)**. Error bars represent the mean ± SEM of three independent replicates. In each replicate, 20 female adult flies were used for each experimental condition. LP treatment had no effect on survival [Mantel-Cox; PBS: LP (1:2), *P* = 0.0811; PBS: LP (1:5), *P* = 0.0551], nor were there differences between LP and CLD treatments [Mantel-Cox; LP (1:2):CLD (1:2), *P* = 0.5506; LP (1:5):CLD (1:5), *P* = 0.6947]. Following perfusion two-days post-injection, the percentage of mCherry^+^ hemocytes were evaluated in LP- and CLD-treated flies (1:5 dilution) **(C)**. Data represent the pooled mean ± SEM of three independent experiments and were analyzed by a Mann–Whitney test to determine significance. To further validate phagocyte depletion, *mCherry* expression was examined in whole flies by qRT-PCR **(D)**. Relative *mCherry* transcripts were significantly reduced following CLD-treatment. Data represent the pooled mean ± SEM of four independent experiments and were analyzed using an unpaired t test to determine significance. n = number of individual flies examined. Asterisks denote significance (***P < 0.001, ****P < 0.0001).

To determine the applicability of using CLD to deplete phagocytic cell populations in other insect species (Kwon and Smith, 2019), we first examined the use of CLD in *Drosophila melanogaster*. Following the injection of either LP (empty) or CLD at different dilutions (1:2 or 1:5 in 1× PBS, Figure 1A), adult *Drosophila* (SRP-mCherry) were monitored over an 8-day period to examine the potential effects of liposome treatment on fly survival (Figure 1B). When compared to PBS-injected controls, LP treatment had no effect on survival [Mantel-Cox; PBS: LP (1:2), *P* = 0.0811; PBS: LP (1:5), *P* = 0.0551] (Figure 1B). In addition, no differences in *Drosophila* survival were seen between LP and CLD treatments [Mantel-Cox; LP (1:2):CLD (1:2), *P* = 0.5506; LP (1:5):CLD (1:5), *P* = 0.6947] (Figure 1B). Using the 1:5 dilutions of LP and CLD, we then evaluated the efficacy of phagocyte depletion by perfusing flies two days post-injection (Figure 1A). Taking advantage of transgenic stocks that express fluorescent proteins in phagocytic plasmatocyte populations (Zettervall et al., 2004; Gyoergy et al., 2018), we demonstrate that CLD treatment significantly reduces the percentage of mCherry^+^ (Figure 1C) and EGFP^+^ (Supplementary Figure 1) plasmatocytes in *Drosophila* adults. We further validated these depletion experiments in the SRP-mCherry line using qRT-PCR, demonstrating a significant reduction in *mCherry* expression 24 h after CLD treatment (Figure 1D). Similar qRT-PCR experiments with the *Hemese*Gal4-UAS-GFP line did not display differences in *GFP* expression when evaluated 24 h post-treatment or at 48 h post-treatment to allow at additional incubation time (Supplementary Figure 1). Given the reduction of EGFP^+^ immune cells following clodronate treatment (Supplementary Figure 1), the lack of change to *GFP* expression levels may be due to *GFP* expression in other tissues beyond plasmatocyte populations as previously noted (Zettervall et al., 2004). Together, these data suggest that CLD are able to effectively deplete *Drosophila* phagocyte populations.

**FIGURE 2:**
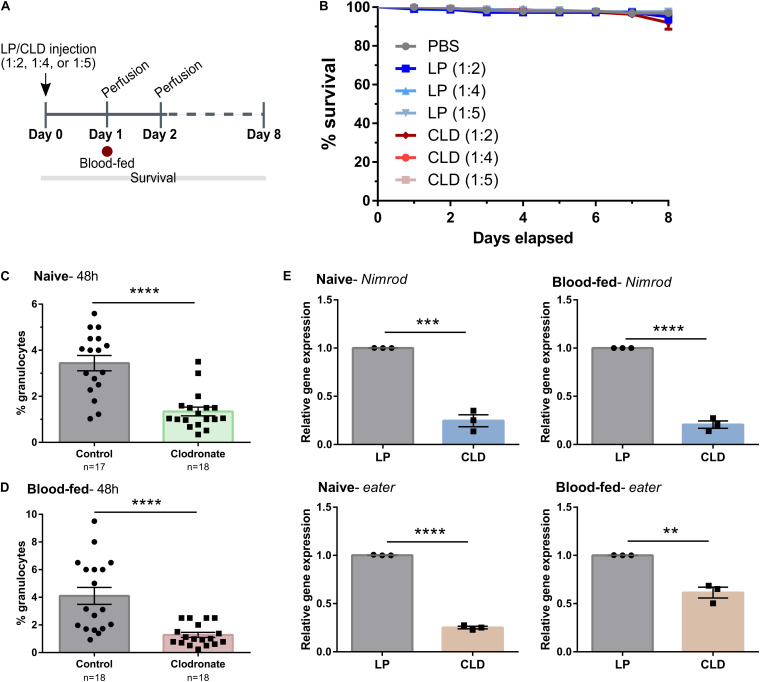
Application of clodronate liposomes to deplete *Ae. aegypti* phagocytic immune cells. **(A)** Overview of clodronate liposome experiments in mosquitoes. Control (LP)- or clodronate liposomes (CLD) were diluted at 1:2, 1:4, or 1:5 in 1X PBS and intrathoracically injected into adult female mosquitoes. Survival was then monitored over an eight-day period **(B)**. Error bars represent the mean ± SEM of two independent replicates. In each replicate, 30 adult female adult mosquitoes were used for each experimental condition. LP treatment had no effect on survival [Mantel-Cox; PBS: LP (1:2), *P* = 0.4464; PBS: LP (1:4), *P* = 0.7120; PBS: LP (1:5), *P* = 0.7978] and there were no differences in survival between LP and CLD treatments [Mantel-Cox; LP (1:2):CLD (1:2), *P* = 0.9425; LP (1:4):CLD (1:4), *P* = 0.7992; LP (1:5):CLD (1:5), *P* = 0.8361]. To evaluate phagocyte depletion, the percentage of granulocytes were examined by light microscopy two-days (48 h) post-injection (1:4 dilution) under either naïve **(C)** or blood-fed conditions **(D)**. Data represent the pooled mean ± SEM of three independent experiments that were analyzed by a Mann–Whitney test to determine significance. To further validate phagocyte depletion, molecular marker of phagocytic cells, *nimrod* and *eater*, were examined in whole mosquitoes by qRT-PCR **(E)**. Relative *nimrod* and *eater* transcripts were significantly reduced following CLD-treatment under both naïve and blood-fed conditions. Data represent the pooled mean ± SEM of three independent experiments that were analyzed using an unpaired t test to determine significance. *n* = number of individual mosquitoes examined. Asterisks denote significance (**P < 0.01, ***P < 0.001, ****P < 0.0001).

Similar experiments were also performed in the yellow fever mosquito, *Aedes aegypti*, to evaluate the use of CLD for phagocyte depletion (Figure 2A). Concentrations of either LP or CLD at 1:2, 1:4, or 1:5 dilutions were examined, with none of the concentration having measurable impacts on adult mosquito survival (Figure 2B). Both the 1:4 and 1:5 dilutions were able to significantly reduce the percentage of granulocytes at 24- or 48-h post-treatment (Supplementary Figure 2), although phagocyte depletion was more effective at 48 h and with the 1:4 dilution (Supplementary Figure 2). Moreover, CLD treatment was able to effectively reduce phagocyte populations in mosquitoes under both naïve (Figure 2C) and blood-fed conditions (Figure 2D). These morphological observations were further validated using qRT-PCR on *eater* and *nimrod*, two transcripts associated with hemocyte phagocytic function (Kocks et al., 2005; Kurucz et al., 2007; Kwon and Smith, 2019). For both *eater* and *nimrod*, clodronate treatment significantly reduced the relative transcript abundance in naïve and blood-fed mosquitoes (Figure 2E). Together, these data suggest that CLD can effectively be used to study *Ae. aegypti* phagocyte function.

**FIGURE 3:**
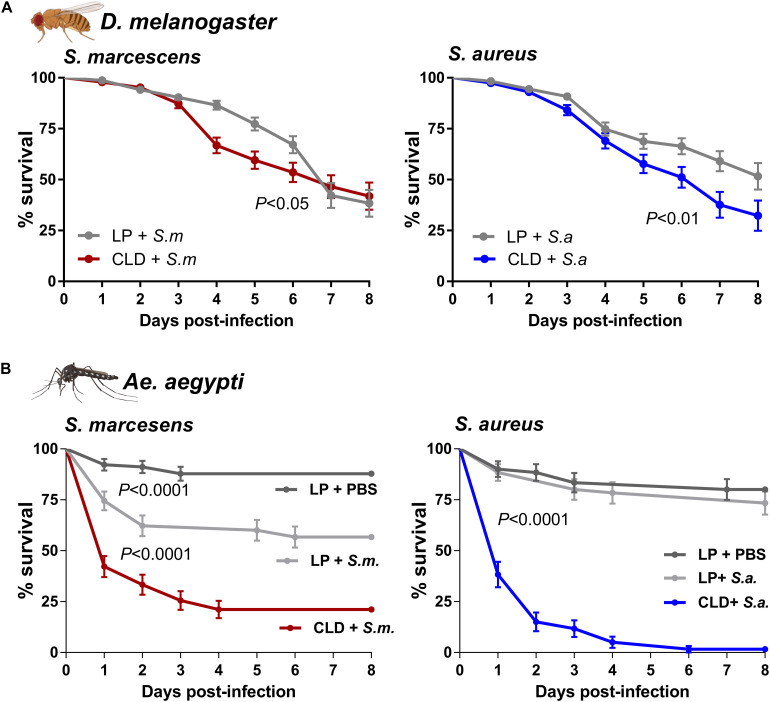
The depletion of phagocytic immune cells influences survival after bacterial challenge. Bacterial challenge assays were performed in flies **(A)** or mosquitoes **(B)** following treatment with control (LP)- or clodronate liposomes (CLD). Survivorship was monitored in every day over an 8-day period to evaluate the effects of *S. marcescens* or *S. aureus* challenge. For mosquito challenge experiments **(B)**, an additional control was added in which LP-treated mosquitoes were challenged with the injection of sterile PBS. Error bars represent the mean ± SEM of three independent replicates for *Drosophila* (20 per replicate) and *Ae. aegypti* (30 per replicate). Data were analyzed using a log-rank (Mantel-Cox) test using GraphPad Prism 6.0. Fly and mosquito images were created with BioRender.com.

To determine the effects of phagocyte depletion on immune function and host survival, we challenged adult flies and mosquitoes with bacteria after treatment with LP or CLD (Figure 3). *Drosophila* displayed significantly reduced survival following phagocyte depletion when challenged with *S. marcescens* and *S. aureus* (Figure 3A) similar to previous reports in which plasmatocytes were depleted through genetic experiments (Charroux and Royet, 2009; Defaye et al., 2009). However, these effects were considered more moderate when compared to the strong phenotypes resulting from similar experiments in *Ae. aegypti*, where the survival of CLD-treated mosquitoes was severely reduced upon challenge of either *S. marcescens* or *S. aureus* (Figure 3B). Similar to previous work in the mosquito *Anopheles gambiae* (Kwon and Smith, 2019), *S. marcescens* challenge caused significant pathogenicity in control- and clodronate-treated *Ae. aegypti*, although phagocyte depletion led to significant mortality within 3 days post-challenge (Figure 3B). *S. aureus* challenge also led to severe mortality in the phagocyte-depleted background with little effect in control mosquitoes (Figure 3B). In agreement with previous studies implicating phagocytic immune cells in mediating insect responses to bacterial challenge (Kocks et al., 2005; Kurucz et al., 2007; Hashimoto et al., 2009; Kwon and Smith, 2019), these results provide further support that CLD can serve as a valuable tool to study cellular immune function and phagocyte contributions to innate immune responses to pathogens across a variety of insect systems.

## Discussion

Insects have developed a robust innate immune system for defense against a variety of microorganisms that are the result of developments in diverse ecological systems and environments, as well as the hematophagous behaviors that expose many insect species to bacterial, viral, fungal, and parasitic pathogens. With evidence of immune memory (Pham et al., 2007; Rodrigues et al., 2010; Cooper and Eleftherianos, 2017) and the conservation of immune signaling pathways with mammalian systems (Buchon et al., 2014; Hillyer, 2016), the study of insect immunity offers several advantages for comparative immunology. Moreover, insects have integral roles in the transmission of disease that influence agriculture or that are of veterinary or medical importance. While *Drosophila* has served as an excellent model for insect systems, it is not representative of the diversity in insect systems where studies of non-model insects have been limited by the lack of genetic tools.

Herein, we expand on previous reports in *An. gambiae* (Kwon and Smith, 2019) to describe the use of CLD in *D. melanogaster* and *Ae. aegypti* to deplete phagocytic immune cells. Widely used in mammalian systems to deplete macrophage populations function (van Rooijen and Sanders, 1994; Lehenkari et al., 2002; van Rooijen and Hendrikx, 2010), our results provide further evidence that CLD can also be utilized in a variety of insect systems and is supported by conserved, functional similarities between insect and mammalian phagocytes (Browne et al., 2013).

In our proof of principle experiments, we demonstrate through microscopy and qRT-PCR techniques that CLD can significantly reduce phagocytic plasmatocyte or granulocyte populations respectively in adult *D. melanogaster* and *Ae. aegypti.* While mutations (Braun et al., 1997, 1998) or other methods of genetic ablation (Charroux and Royet, 2009; Defaye et al., 2009) to study phagocyte function already exist in *Drosophila*, similar tools have not yet been developed in mosquitoes. Alternative methods to inhibit phagocyte function have been utilized in both *Drosophila* (Elrod-Erickson et al., 2000; Lamiable et al., 2016) and mosquitoes (Castillo et al., 2017) that rely on saturating the phagocytic machinery via the injection of polystyrene beads, yet may not fully impair phagocyte function. Therefore, we believe that the use of CLD provides a convenient method to study phagocyte function in non-model insects, as well as an alternative methodology for model systems such as *Drosophila*. Moreover, the ability to deplete phagocytic cell populations also enables the study of phagocyte contributions to insect-pathogen interactions. This is supported by recent experiments demonstrating phagocyte contributions to anti-*Plasmodium* immunity in *An. gambiae* (Kwon and Smith, 2019) and may be similarly utilized in the future to examine phagocyte function in the context of arbovirus infection in *Ae. aegypti*.

Additional experiments demonstrate the importance of phagocyte function for insect survival following bacterial challenge, wherein both flies and mosquitoes display reduced survival to gram (−) and gram (+) bacteria following phagocyte depletion similar to previous experiments (Charroux and Royet, 2009; Defaye et al., 2009; Kwon and Smith, 2019). Of interest, these survival phenotypes were much stronger in *Ae. aegypti* where few mosquitoes survived challenge with either *S. marcescens* or *S. aureus*, and may potentially represent differences in the cellular and humoral defenses to bacterial pathogens between mosquitoes and flies that warrant further study.

In summary, we believe that our experiments with CLD support their utility to deplete phagocytes in flies and mosquitoes, providing new or alternative methods to study the cellular and humoral contributions of phagocytes to the defense of invading pathogens. With the conserved utility of CLD in mammals and insects, as well as its ease of use, we believe that CLD can be a significant new resource for the study of invertebrate immunity.

## Data Availability Statement

The original contributions presented in the study are included in the article/Supplementary Material, further inquiries can be directed to the corresponding author.

## Author Contributions

JR, JS, and HK performed the experiments and analyzed the results. RS conceived the experiments, analyzed data, and wrote the initial draft of the manuscript. All authors contributed to the editing and writing of the final manuscript.

## Conflict of Interest

The authors declare that the research was conducted in the absence of any commercial or financial relationships that could be construed as a potential conflict of interest.
