# Supplementary material for: Use of Clodronate Liposomes to Deplete Phagocytic Immune Cells in Drosophila melanogaster and Aedes aegypti
Source: Front Cell Dev Biol. 2021 Feb 2;9:627976. doi: 10.3389/fcell.2021.627976 (PMC7884637; doi:10.3389/fcell.2021.627976)
Supplement: Supplementary file 1 [file Image_1.pdf]

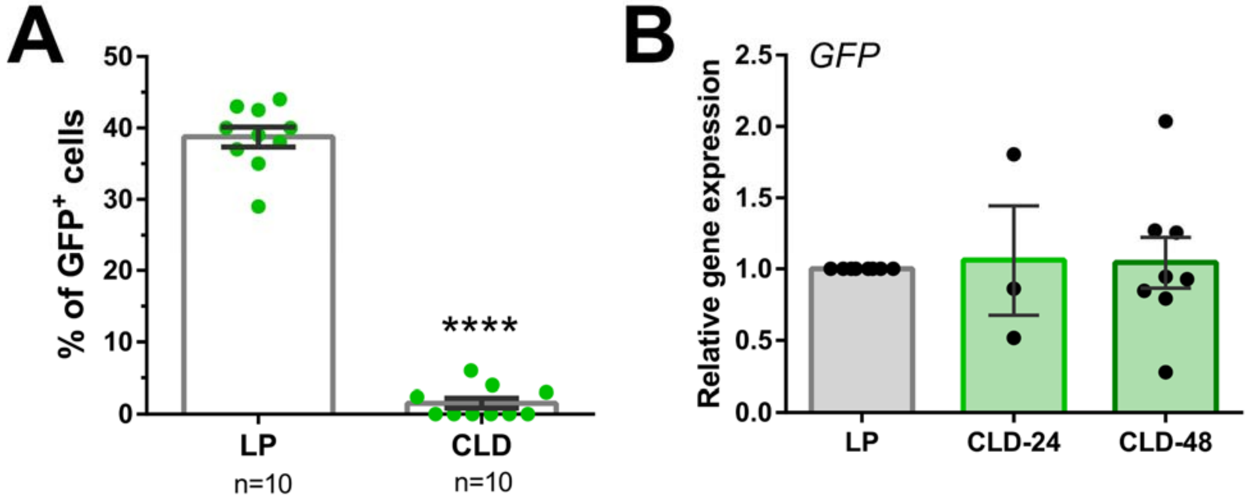

Figure S1. Clodronate liposome treatment to deplete *Drosophila* plasmatocytes in the *Drosophila* HeGal4-UAS-GFP line. Control (LP)- or clodronate liposomes (CLD) were diluted at 1:5 in 1X PBS and intrathoracically injected into adult female flies. Following perfusion two-days post-injection, the percentage of GFP^+ hemocytes were evaluated in LP- and CLD-treated flies **(A)**. Data represent the pooled mean ± SEM of two independent experiments and were analyzed by a Mann–Whitney test to determine significance. As an additional method to evaluate phagocyte depletion, *GFP* expression was examined in whole flies by qRT-PCR **(B)**. No effects on relative *GFP* transcripts were found following CLD-treatment at either 24- or 48-hours post-injection. Data represent the pooled mean ± SEM of three or more independent experiments and were analyzed using a one-way ANOVA and Holm-Sidak multiple comparison test to determine significance. n = number of individual flies examined. Asterisks denote significance (****$P < 0.0001$).
