# Supplementary material for: Use of Clodronate Liposomes to Deplete Phagocytic Immune Cells in Drosophila melanogaster and Aedes aegypti
Source: Front Cell Dev Biol. 2021 Feb 2;9:627976. doi: 10.3389/fcell.2021.627976 (PMC7884637; doi:10.3389/fcell.2021.627976)
Supplement: Supplementary file 2 [file Image_2.pdf]

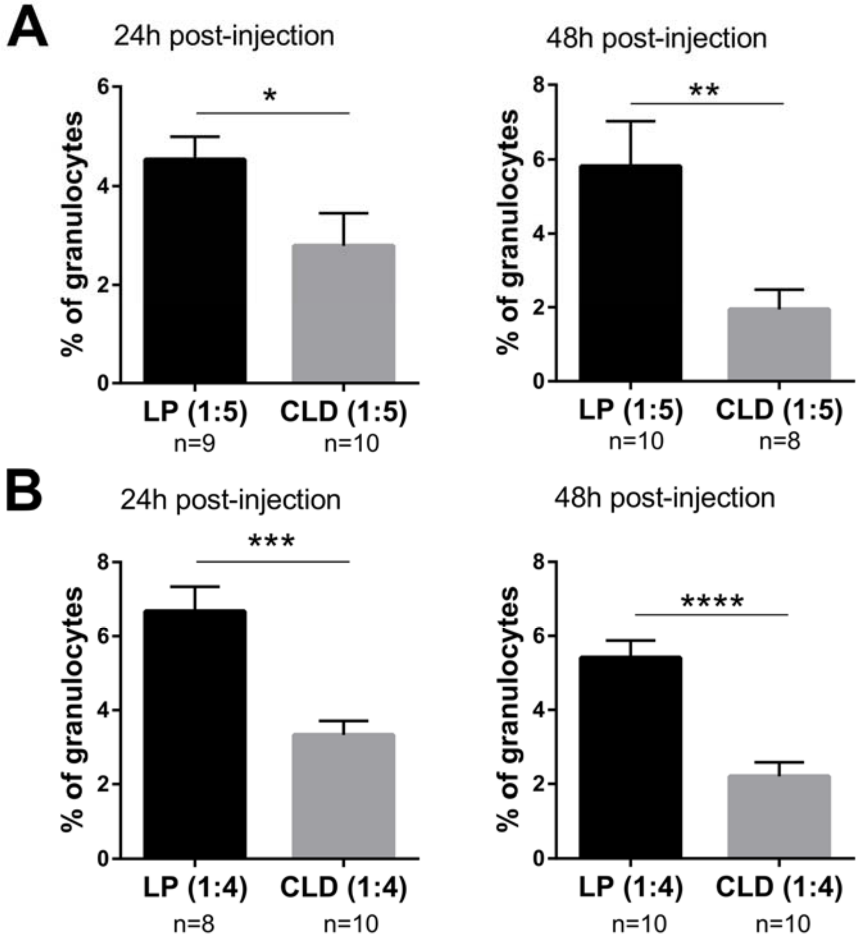

Figure S2. Influence of clodronate liposome dilutions and timing post-injection on granulocyte depletion in *Ae. aegypti*. Clodronate liposome treatments to deplete granulocytes in *Ae. aegypti* were evaluated 24- or 48-h post injection using the intrathoracic injection of a 1:5 **(A)** or 1:4 dilution in 1X PBS **(B)**. Following perfusion, the percentage of granulocytes (as identified by morphology) were evaluated in control (LP)- and clodronate liposome (CLD)-treated mosquitoes. Data represent the pooled mean ± SEM of two independent experiments and were analyzed by a Mann–Whitney test to determine significance. n = number of individual flies examined. Asterisks denote significance (*$P < 0.05$, **$P < 0.01$, ***$P < 0.001$, ****$P < 0.0001$).
