# Supplementary material for: Use of Clodronate Liposomes to Deplete Phagocytic Immune Cells in Drosophila melanogaster and Aedes aegypti
Source: Front Cell Dev Biol. 2021 Feb 2;9:627976. doi: 10.3389/fcell.2021.627976 (PMC7884637; doi:10.3389/fcell.2021.627976)
Supplement: Supplementary file 3 [file Table_1.docx]

**Table S1. Primers for qRT-PCR analysis**

**Primer Gene ID Sequence (5’- 3’)**

GFP-F N/A CCATGGCCAACACTTGTCACTA

GFP-R CGTGTCTTGTAGTTCCCGTCATC

mCherry-F N/A GGACGGCGAGTTCATCTACAAG

mCherry-R TCTTGACCTCAGCGTCGTAGTG

*Dm* RpL32-F CG7939 GACGCTTCAAGGGACAGTATCTG

*Dm* RpL32-R AAACGCGGTTCTGCATGAG

*Aa* Nimrod-F AAEL019650 TGCTTGAAGTCCGGTCTGGGTCAAGAAGGTGT

*Aa* Nimrod-R GGTTTCACCAGAGTAATGGTTGGCACCTCTTCG

*Aa* eater-F AAEL000636 TGTTTTAGACGACGGTTCCTGTCAAT

*Aa* eater-R GCTTCAGTTTTTCGATAGCCATCCT

*Aa* rpS17-F AAEL004175 TGGTTTCGTGACACATCTGATGAAG

*Aa* rpS17-F AGCTGCTTCAACATCTCCTTGGTCT
